# Precision Agriculture Design Method Using a Distributed Computing Architecture on Internet of Things Context †

**DOI:** 10.3390/s18061731

**Published:** 2018-05-28

**Authors:** Francisco Javier Ferrández-Pastor, Juan Manuel García-Chamizo, Mario Nieto-Hidalgo, José Mora-Martínez

**Affiliations:** Department of Computer Technology, University of Alicante, P.O. Box 99, E-03080 Alicante, Spain; juanma@dtic.ua.es (J.M.G.-C.); mnieto@dtic.ua.es (M.N.-H.); jose.mora.martinez.es@gmail.com (J.M.-M.)

**Keywords:** Internet of Things, precision agriculture, fog and edge computing

## Abstract

The Internet of Things (IoT) has opened productive ways to cultivate soil with the use of low-cost hardware (sensors/actuators) and communication (Internet) technologies. Remote equipment and crop monitoring, predictive analytic, weather forecasting for crops or smart logistics and warehousing are some examples of these new opportunities. Nevertheless, farmers are agriculture experts but, usually, do not have experience in IoT applications. Users who use IoT applications must participate in its design, improving the integration and use. In this work, different industrial agricultural facilities are analysed with farmers and growers to design new functionalities based on IoT paradigms deployment. User-centred design model is used to obtain knowledge and experience in the process of introducing technology in agricultural applications. Internet of things paradigms are used as resources to facilitate the decision making. IoT architecture, operating rules and smart processes are implemented using a distributed model based on edge and fog computing paradigms. A communication architecture is proposed using these technologies. The aim is to help farmers to develop smart systems both, in current and new facilities. Different decision trees to automate the installation, designed by the farmer, can be easily deployed using the method proposed in this document.

## 1. Introduction

**Figure 1:**
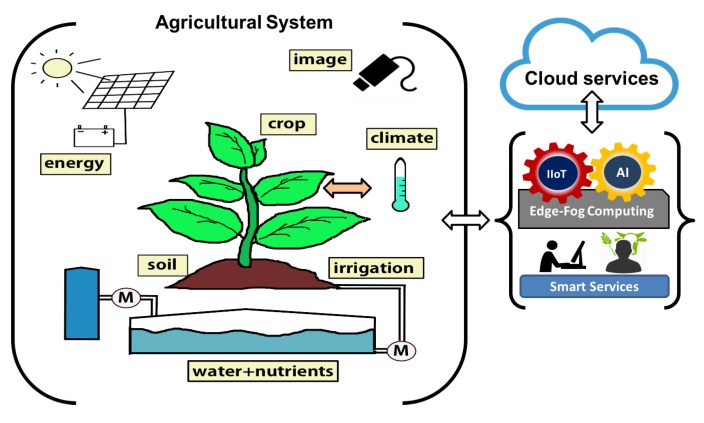
Agricultural System.

Precision Agriculture (PA) is a whole-farm management approach using information technology, remote sensing and proximal data gathering. These technologies have the goal of optimising returns on inputs while potentially reducing environmental impacts. Farmers and agronomists have already begun employing technologies in order to improve the efficiency of their work. Sensors placed in greenhouses allow farmers to obtain detailed data on real-time as variables such as soil and ambient temperature, irrigation water and soil conductivity, soil and irrigation water PH, nutrient composition data, irrigation water properties, etc. These data can be transmitted and analysed using communication technologies and Artificial Intelligence (AI) paradigms could be applied. Farmers use their smartphones to remotely monitor their crops and equipments and to run some statistical data. All of these techniques help make up PA. Nowadays, the farmers are using resources developed by information and communication technologies. These first resources are easy to use but they are general purpose and, therefore, not adapted to the specific needs of each farmer. In this work an user-centred method is proposed to design intelligent and adapted services where each farmer decides its own installation using edge and fog paradigms (distributed computing) on Internet of Things technologies (Figure 1). This method is designed on different use cases and tested in an automated greenhouse as an example of utility. The work is an expanded version presented in [1]. This paper is organized as follows: Section 2 reviews precision agriculture scenarios, how to use centred design methodologies, IoT technologies and their deployment, the capabilities and potential of edge and fog computing paradigms in these scenarios. Different greenhouses are analysed and farmers are consulted. Section 3 proposes a method to deploy distributed IoT architecture using edge and fog nodes that offer a set of new resources that can be used in any type of installation which facilitates the involvement of the farmer. In Section 4, experiments including services on edge and fog nodes, connected by IoT communication protocols, are performed. Finally, Section 5 provides conclusions and future works.

## 2. Related Works

Although more complex definitions exist, the simple description of the PA is a way to “apply the right treatment in the right place at the right time” [2]. Precision agriculture comprises a set of technologies that combine sensors, information systems, enhanced machinery and informed management to optimize production by accounting for variability and uncertainties within agricultural systems. It is a farming management concept based upon observing, measuring and responding to inter and intra-field variability in crops [3]. Methods and technologies to decide how, where and when to use sensors and machinery should involve all the main actors: that is, farmers and information and communication technicians. User-centred methods and IoT communication technologies applied on precision agriculture are revised in this section. Finally, different greenhouses are analysed and farmers are consulted.

### 2.1. User-Centred Design Models

In PA context, User-Centred Design (UCD) describe a design process where farmers influence the process of how the design takes shape. There are several ways in which the user (agricultural specialist) can be involved in the process. This term describes a set of methods to create models on which design adapted solutions. The user-centred design process works against subjective assumptions about user behaviour. It requires proof that the design decisions are effective. If user-centred design is properly done, applications becomes an outcome of actively engaging users. Therefore, any design decisions that were made by observing and listening to them will not be based on personal preferences. User experience (UX) is one of the many focuses of UCD. It includes the user’s entire experience with the product, including physical and emotional reactions. UCD is objective and often relies on data to support design decisions [4]. According to [5], user centred design is a development method that guarantees that product, software or web site will be easy to use. The International Usability Standard (ISO 13407) [6], specifies the principles that underlie user centred design:

- Requirements gathering, understanding and specifying the context of use
- Requirements specification, specifying the user and organisational requirements
- Design, producing designs and prototypes
- Evaluation, carrying out user-based assessment of the site

The design is based upon an explicit understanding of users, tasks and environments. Users are involved throughout design and development. The process is iterative. The design is driven and refined by user-centred evaluation. The design addresses the whole user experience. The design team includes multidisciplinary skills and perspectives.

### 2.2. Internet of Things: Architectures and Protocols

Iot is developed using architectures based on layers capable of connecting a huge number of devices with each other and with the established services. The basic model of IoT has a 3 layer architecture which are of Perception, Network and Application Layers. IoT faces several challenges, especially in the field of privacy and security, so to overcome these issues new standard architectures need to be more focused on many essential factors like Quality of Services (QoS), data integrity, sustainability, confidentiality, etc. The IAB (Internet Architecture Board) has published the RFC 7452 document: Architectural Considerations in Smart Object Networking. This document offers guidance to engineers designing Internet-connected smart objects. A Request for Comments (RFC), in the context of Internet governance, is a type of publication from the Internet Engineering Task Force (IETF) and the Internet Society (ISOC), the principal technical development and standards-setting bodies for the Internet. Table 1 illustrates different works that apply the layer model in the IoT architecture.

IoT needs protocols adapted to the new requirements. Traditional protocols are extended and new protocols are proposed offering different options on different contexts. IoT has now a wide range of applications. A smart devices can have wired or wireless connection. As far as the wireless IoT is the main concern, many different wireless communication technologies and protocols can be used to connect the smart device such as Internet Protocol Version 6 (IPv6), over Low power Wireless Personal Area Networks (6LoWPAN), ZigBee, Bluetooth Low Energy (BLE), Z-Wave and Near Field Communication (NFC). They are short range standard network protocols, while SigFox and Cellular are Low Power Wide Area Network (LPWAN).standard protocols. In [11] a review and comparison of different communication protocols in IoT is realised. This comparison aims at presenting guidelines for the researchers to be able to select the right protocol for different applications. Table 2 illustrates different protocols used in the architecture layers.

Choosing the most appropriate protocol depends on several facts of which most important are: environmental conditions, network characteristics, the amount of data to be transferred, security levels and quality of service requests [12]. CoAP network is primarily a one-to-one protocol for transferring state information between client and server while MQTT is a many-to-many communication protocol for exchanging messages between multiple clients. CoAP runs over UDP which means that communication overhead is significantly reduced. If constrained communication and battery consumption is not an issue, RESTful services can be easily implemented and interact with the Internet using the worldwide HTTP [13]. If the targeted final applications require massive updates of the same value, MQTT protocol is more suitable. In this work different protocols (MQTT, HTTP, Bluetooth, WiFi, LTE, ...) can be used to develop proposed architecture.

### 2.3. Internet of Things Technologies Applied on PA Scenarios

Advance in electronics, computing and telecommunications are allowing the development of new devices (sensors, actuators and computing nodes) with wireless communication capabilities, installed at any location, smaller, energy efficient, autonomous, more powerful and low cost [14,15,16,17,18]. IoT works using user-driven service modeling is proposed in [19]. Low-cost IoT devices that need to gather and transmit sensor data and receive remote commands is shown in [20,21,22]. IoT uses the connection between devices to improve their efficiency and user experience, being the communication one of the main elements for a proper IoT network. A review of the most common wired and wireless communication protocols, discussion of their characteristics, advantages and disadvantages as well as a comparison study to choose the best bidirectional sensor network composed by low power devices is realised in [23]. Previous works show the degree of development of IoT technology, which has also been experienced in precision agriculture in recent years.

IoT technologies are proposed in PA scenarios. In [24] this paradigm is analysed as a solution in precision farming. IoT Smart farming application include farm parameters tracking, monitoring, field observation and storage monitoring. The work Internet of Things Platform for Smart Farming [25] presents a platform based on IoT technologies that can automate the collection of environmental, soil, fertilisation, and irrigation data; automatically correlate such data and filter-out invalid data from the perspective of assessing crop performance; and compute crop forecasts and personalised crop recommendations for any particular farm. This platform (SmartFarmNet) can integrate virtually any IoT device, including commercially available sensors, cameras, weather stations, etc., and store their data in the cloud for performance analysis and recommendations. An evaluation of the SmartFarmNet platform and the experiences and lessons learnt in developing this system concludes the paper. SmartFarmNet is the first and currently largest system in the world (in terms of the number of sensors attached, crops assessed, and users it supports) that provides crop performance analysis and recommendations.

In [9] a greenhouse with hydroponic crop production was designed, developed and tested using Ubiquitous Sensor Network monitoring and control on Internet of Things paradigm. The experimental results showed that the Internet technologies and Smart Object Communication Patterns can be combined to encourage development of Precision Agriculture. They demonstrated added benefits (cost, energy, smart developing, acceptance by agricultural specialists) when a project is launched. Other related work is shown in [26] with Zig Bee technology: Artificial intelligence and decision support approaches have been developed. This work develop technology for real-time monitoring of citrus soil moisture and nutrients and the research on the integration of fertilization and irrigation decision support system. The results showed that the system could help the grower to scientifically fertilize or irrigate, improve the precision operation level of citrus production, reduce the cost and reduce the pollution caused by chemical fertilizer. A review into the state-of-the-art of Big Data applications in Smart Farming is performed in [27]. Malche et al. [28] proposed a prototype IoT system for water level monitoring which can be implemented in future smart villages in India. Manufacturers of the agricultural sector highlights the importance of IoT in [29,30,31,32]. PA is effectively a suite of methods, approaches and instrumentation that farmers should examine in detail to decide which is the most suitable for their business.

### 2.4. Internet of Things, Cloud and Machine Learning Evolution: Edge and Fog Computing Paradigms

Internet of Things (IoT) aims to bring every object (e.g. smart cameras, environmental sensors, control appliances, machine learning analysis) on line, hence generating massive amounts of data that can overwhelm storage systems and data analytic applications. Cloud computing offers services at the infrastructure level that can scale to IoT storage and processing requirements. However, there are applications such as sensor monitoring, control and analysis response that require low latency therefore, delay caused by transferring data to the cloud and then back to the application can seriously impact their performances. To overcome this limitation, Fog and Edge computing paradigms have been proposed, where cloud services are extended to the edge of the network to decrease the latency and network congestion [33]. Both fog computing and edge computing involve pushing intelligence and processing capabilities down closer to where the data originates from pumps, motors, sensors, relays, etc. The key difference between the two architectures is exactly where that intelligence and computing power is placed:

- Fog computing pushes intelligence down to the local area network level, processing data in a fog node or IoT gateway
- Edge computing pushes the intelligence, processing power and communication capabilities of an edge gateway or appliance directly into devices like programmable automation controllers (PACs)

With IoT implementation now becoming more widespread, devices will generate a lot of data at the end of the network and many applications will be deployed at the edge to process the information. Cisco Systems predicts that an estimated 50 billion devices will connect to the Internet by 2020 [34,35,36]. Some of the applications they run might require very short response times, some might involve private data, and some might produce huge quantities of data. Cloud computing cannot support these IoT applications. Edge and Fog computing paradigms, on the other hand, can do so and will promote many new IoT applications.

The work done in [37] concludes that the wireless sensor and actuator networks based on Edge computing are experiencing fast development and opportunities in the post-Cloud era, and are used in more and more applications. In [38] a Fog Computing Based on radio access networks is proposed for smart-cities services.

### 2.5. Automated Technologies in Greenhouses

Different greenhouses in the south east of Spain have been visited to analyse the type of installation and to ask expert users. The greenhouse with the highest level of automation showed a complete number of sensors and actuators; however, not all the sensors could be related. There are two large subsystems in self-assembled greenhouses that are not interoperable. These subsystems install different types of control and technologies:

- Irrigation and nutrition
- Air conditioning and ventilation.

**Figure 2:**
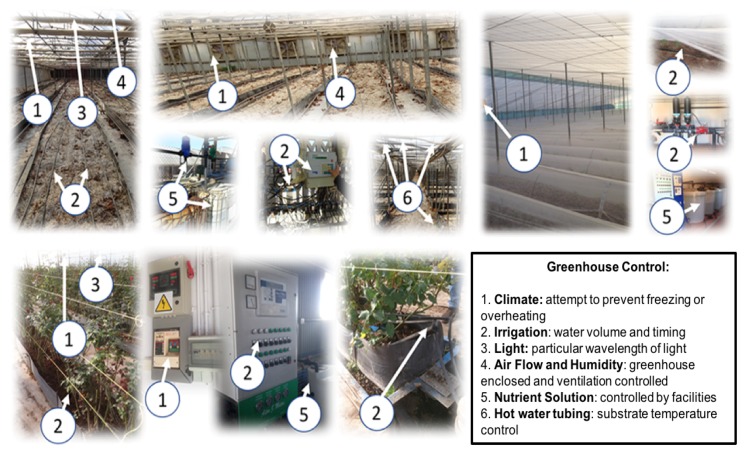
Automated greenhouses: main subsystems in current facilities.

In these facilities, an ambient temperature sensor of the air conditioning system is not related to an irrigation water temperature sensor of the irrigation system. Figure 2 shows different automated greenhouses where main subsystems are listed.

## 3. User Centred and Computing Method Model: Distributed Computing Architecture Based on Edge and Fog Nodes

The current agricultural facilities are divided in subsystems (irrigation, light, climate, soil, crop and energy) that are not interconnected. Industrial logic programmable controllers and specialised sensors give basic automation services in each subsystem. Internet and electronic devices (smartphones) provide new functionalities based on information access, control and monitoring. Human interfaces on smartphones connected to web servers are examples of new services developed over the past years. Agricultural technician and farmers have knowledge that can be converted on expert rules for device control. These rules are programmed and implemented on actual programmable devices; however, they are static rules which means that do not evolve when new conditions occur, neither do they adapt to the singularities of each installation. The farmer has to decide how to set the rules: what pH the irrigation water must have, how much water should be programmed in irrigation process, etc. Also, each rule only has effects in a subsystem (irrigation, climate), there is no interoperability.

**Figure 3:**
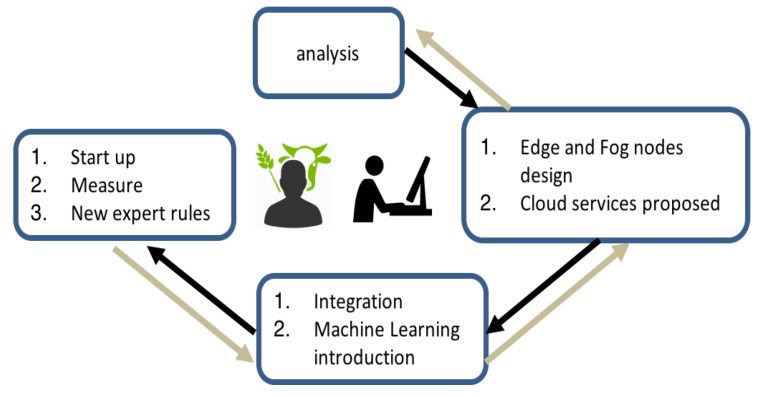
User centred model based on design and integration of edge and fog communication levels. Cloud services and machine learning processes are integrated using this method.

Considering this context, new facilities design and development method are proposed in this work. The aim is that the farmer participates in the automated activities and that the subsystems become interoperable. A method that implement automatic rules and automate the decision making considering the behaviour of the installation itself are also proposed. The phases of the proposed model are shown in Figure 3.

**Figure 4:**
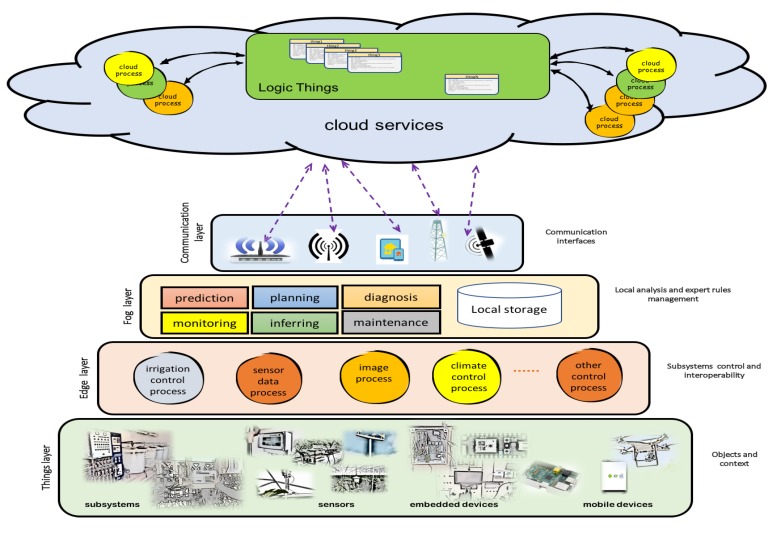
Architecture: communication levels with different functionality.

- Analysis: two kind of users are identified in this phase (agriculture user expert and ICT technician). Expert users in agriculture are interviewed to define main processes to control. All these issues are related with ICT expert in a participatory design. The results of this first approach are the things required to design services and control. In this phase an user-centred methodology captures the farmer requirements.
- Design: the model is based on an architecture with three levels: edge, fog and cloud services. In this phase an adapted architecture using these levels is designed. The adapted architecture is shown in Figure 4.
- Integration and data analysis: Installation and Integration subsystems are developed in this phase. Data analysis is proposed to design machine learning services based on expert rules with farmer.
- Start up, measure and feedback: Test and feedback are launched. The first expert rules are integrated with farmer supervision. New rules are designed with feedback processes. Automatic and adapted rules are developed using artificial intelligence systems with machine learning platforms.

### 3.1. User-Centred Analysis and Design

There are two cases treated:

- Agricultural installation with some automated facilities already installed
- New agricultural installation

The method is the same for both. Expert users in agriculture are interviewed to define new processes to control. In this first approach, the things (objects) required, their relationships and the potential services are detected. Once objects and services have been detected, they must be related to the necessary communication and control technologies (IoT protocols). Human Interfacing are adjusted. Expert rules and intelligent services are analysed (Edge and Fog computing proposal). Finally, the installation, maintenance and operation methods are designed. All this is designed between user agricultural technician and information technologies expert.

The results of this first approach are the *things* required to design services and control. A first set of sensors, actuators, variables, processes and controllers are designed considering production facility. This set of objects will be considers like *things* in the next stage.

In this description, a *thing* is formed by an object/entity and a context with data associated.

Each *thing* has a n-tuple data structure (ID,time,date,location,relations,state) where ID, data, location, relations with other things and states are defined. Table 3 represents different *things*. Expert users design control rules using the things defined. These control rules are part of control processes (climate, soil, irrigation, crop, energy or image) that are distributed in different embedded systems connected to the network (intranet/internet). Things are a virtual representation of all available resources that can be deployed in the different subsystems of the installation.

At this level all objects/things are recognized by designers. Basic control algorithms of all subsystems are designed.

### 3.2. Integration: Architecture Development

In the previous phase objects and their relationship with basic algorithms has been designed. An architecture adapted to the facility available is developed in this phase. Requirements are:

1. Interconnection and data access of all subsystems data
2. Facilities and resources to implement expert rules
3. Configuration, operation and modification processes

IoT and AI paradigms provide resources to propose an innovative architecture that can be used in new smart precision agriculture services. Edge computing used on control devices and fog computing nodes installed on local network provide powerful technologies to implement configuration, operation and improvement processes.

IoT protocols provide resources to capture and communique all subsystems data. Each subsystem is composed by objects/things (sensor/actuator) that can be connected and processed using nodes on sensor networks with IoT protocols. The requirements established for PA scenarios are:

- Standard and interoperable communication protocols to develop open and reusable applications.
- Low-power consumption of all devices installed improve the establishment and development.
- Easy access and maintenance. Users are not specialized in information technologies. It is important to improve its acceptability.
- Support the integration of new smart modules (modularity and scalability software and hardware).
- Non-proprietary hardware-software to reduce dependencies.
- Low cost devices selection increases the level of penetration.

IoT protocols are designed to work on communication scenarios and requirements established. They are optimised for control and two-way open communication channels. In these works [39,40,41] Message Queuing Telemetry Transport (MQTT) protocol is proposed as communication paradigm between sensors, actuators, communication nodes, devices and subsystems. Some of the features that makes it especially suitable for this project are:

- MQTT is a publish-subscribe messaging protocol developed for resource-constrained devices [42], a model already in use by enterprises worldwide, and can work with legacy systems.
- All messages have a topic path composed of words separated by slashes. A common form is /place/device-type/device-id/measurement-type/status. The subscribers may use wild-cards to subscribe to all measurements coming from a specific class of device.
- The bandwidth requirements are extremely low, and the nature of the protocol makes it very energy-efficient.
- The programming interface is very simple, and the client memory footprint is small, making it especially suitable for embedded devices.
- Three Quality of Service (QoS) levels provide reliable operations [43].

Ubiquitous networks allow an *n-to-m* nodes communication model. Any node is able to query and be queried by other nodes. In addition, any node may play the role of a base station (skin node) capable of transmitting its information to remote processing places using a gateway device. USN local nodes can use and process local data, with a gateway these nodes have a global accessibility and they offer extended services on an IoT scenario. Local and global access over the same node (sensor/device/actuator) has different possibilities and benefits. Whereas a local data processing is necessary in basic process control (security, system start-stop, etc.), global processing (analytic) can be used in pattern detection and information generation. In this sense, the proposed platform uses both technologies combined: different USN over a local network area (intranet) connected to cloud-IoT services (internet). A computing layer in local area, called *edge computing*, will serve as interface between control processes and cloud-services. This layer will be able to process data before communicating to cloud.

### 3.3. Data Analysis: Edge and Fog Computing Configuration

The development of edge and fog computing can be understood in three phases:

- Connection: Numerous heterogeneous, real time connections between terminals and devices will serve edge computing, as will automatic network deployment and operation. Additionally, security, reliability, and interoperability of connections should be guaranteed. An application of this phase is remote automatic soil parameters and ambient conditions data reading
- Data treatment on edge computing devices: In this phase, data analysis and automatic services develop new capabilities that are implemented on the new edge nodes. Applications of this phase can be data filtering, predictive calculation of climatic data, classification services or detection events
- Services on fog computing nodes: Enabled by technologies such as AI and IoT communication protocols. Fog computing nodes carries out smart analysis and computing, as well as implementing dynamic, real-time self-optimization, and executing policy adjustments. Applications of this phase are prediction of water consumption, smart detection or unattended production

**Figure 5:**
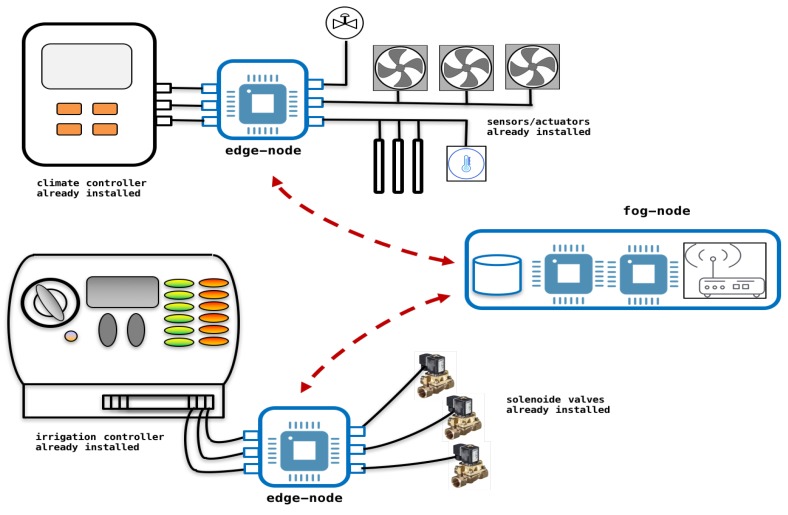
Architecture proposed on facilities already automated: edge nodes interleaved between devices already installed and fog nodes that interconnect all subsystems.

**Figure 6:**
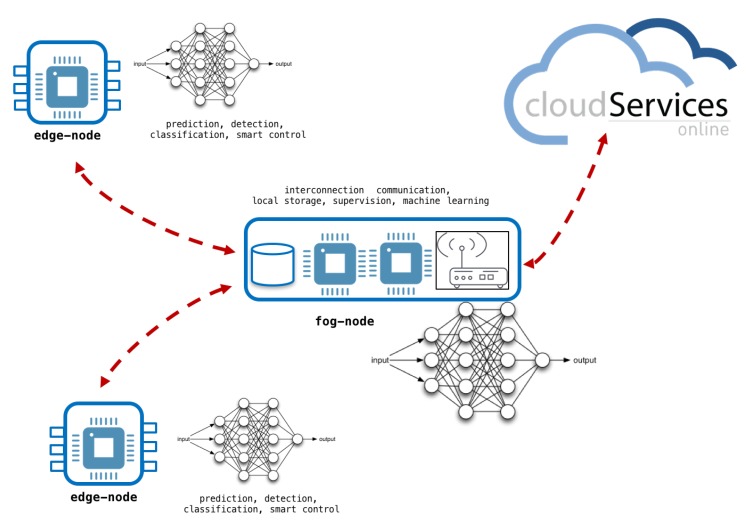
Architecture implemented: services proposed.

Figure 5 shows the architecture implemented using edge and fog nodes. When automated subsystems are already installed it is necessary to interleaved embedded devices (edge-nodes) between controllers and sensors/actuators. This devices maintain the initial services and allow to initiate a supervised learning process. New algorithms are tested and approved on edge and fog nodes. In Figure 6 different services are proposed on each node.

### 3.4. Test and Feedback Developing

In machine learning systems the output is not fixed. It will change over time as the solution knows more and as the model on which the machine learning system is built evolves as it is fed more data. This forces the testing professional to think differently and adopt test strategies that are very different from traditional testing techniques. To test machine learning systems is essential:

1. Obtaining data sets: This refers to a data set with main variables captured and stored to analyse and design the model. In irrigation process data set are: irrigation programming used (time and flow), ambient conditions (humidity, temperature) and soil conditions (humidity, temperature, pH and conductivity) captured by sensors. All this data are monitored and stored.
2. Training data sets: This refers to a data set used for training the model. It is a subset of the previous dataset. In irrigation process training data set are: irrigation programming automated by the model (time and flow decision) considering ambient conditions (soil, ambient and crop). This data is usually prepared by collecting data in a semi-automated way. The results of this process are validated with agronomists.
3. Testing data sets: It is a dataset used to to measure the model quality.
4. Validation test suites on real scenarios. Taking the irrigation example, test scenarios include categorizing needs of water for a kind of crop considering climatic conditions and its growth phase. Automated irrigation decisions by the model are analysed in this phase.
5. Building validation suites. It is necessary to understand the algorithm. The model has algorithm that analyse the data provided, looks for specific patterns, and uses the results of this analysis to develop optimal parameters for creating the model. The model is refined as the number of iterations and the richness of the data increase.
6. Communicating test results in statistical terms. Models based on machine learning algorithms will produce approximations and not exact results. Quality of results must be analysed in the same context. The testing community will need to determine the level of confidence within a certain range.
7. Model evolution. Support to develop new AI services or modifications on algorithms implemented. Supervised and automatic changes are processes to maintain the operating models.

### 3.5. Comparison with Industrial Facilities. Novelty Elements Proposed

Currently, industrial facilities that use PA technologies are based on integration of internet and web services with automation and control using industrial technology. Proprietary systems are designed for monitoring large production plants. Related work analysed show that the Agriculture Control system for production, irrigation, or climate proposes different monitoring and control technologies, based on wireless sensor network and industrial control. Monitoring systems analyse crop environment and the method to improve the decision making by analysing statistics and reactive algorithms. This work proposes two main novelty elements: optimization of architecture levels integration of edge and fog layers and proposes the integration the farmer in the design of new improvements using data analysis obtained with the new architecture developed.

## 4. Experimental Work

Different agricultural facilities have been analysed to introduce the method proposed. Three kind of installations summarize the different types:

- Installation automated but subsystems not interoperable
- Partial automation without any interconnection and non-interoperable systems
- Manual control

In all of them, the services based on AI are not yet installed. In this context, the method proposed using edge-computing on basic controllers and fog-computing on gateway nodes can design common services for the three types of facilities cited. With this configuration, subsystems interoperability and AI support are achieved. Control signals of already installed controllers in automated installations become inputs to edge nodes and a fog node which acts as an interface for all facility nodes. In all greenhouses, irrigation and internal environment control are basic processes. Agronomist users know how to program reactive controls and how to configure automated devices. Optimization of these resources (water, energy) are two potential services that agronomist perform through their experience. This knowledge can be transferred to intelligent systems that integrate it through techniques based on AI paradigms. Interconnection of subsystems also are one of the proposed improvement. A deployment for an automated installation is designed and implemented. This case shows how to implement when there are already automated installations. This case also serves as a guide for other types of greenhouse installations.

### 4.1. Analysis

The farmer, together with the technician in information technologies, propose a set of improvements:

- Monitoring and control interfaces on the Internet (control and communication services)
- Event and change communication service (communication services)
- Interconnection of irrigation and air conditioning subsystems (interoperability services)
- Integration of automation to optimize water consumption (AI services)

**Figure 7:**
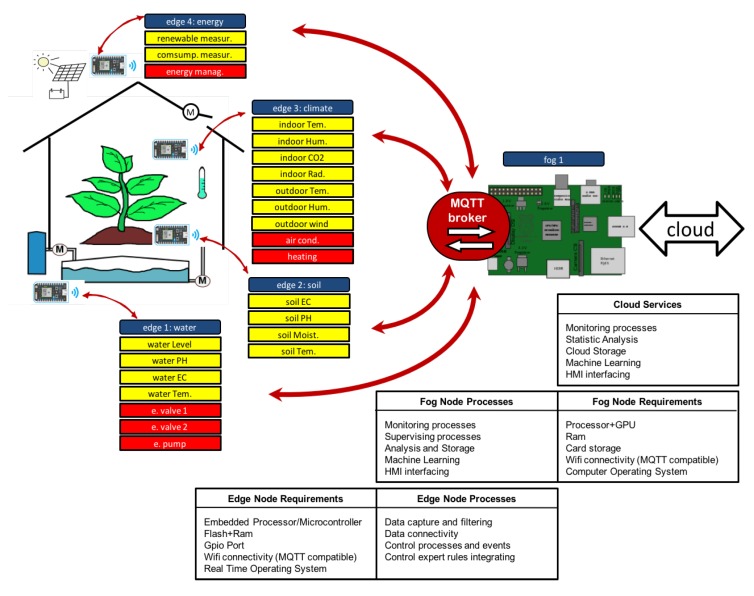
Greenhouse design. Fog and edge nodes relations on agriculture subsystems.

The work carried out designs an installation that deploy the necessary hardware and software resources using the proposed method Figure (Figure 7) shows agricultural subsystems and model deployed on distributed nodes.

Intelligent irrigation control is installed in an experimental greenhouse based on tomato hydroponic cultivation (Table 4). Following the proposed method, the experimental phases are:

1. Things, communication and context design: objects (things) and its context are detected and related using IoT protocols and services (user-centred, architecture and IoT protocols design)
2. Hardware devices and software modules: edge and fog nodes with the services that will be implemented are proposed (integration and AI services)
3. Installing: how the model is tested and deployed (testing process)

### 4.2. Design: Things, Communication and Context

Irrigation process, soil parameters, environmental conditions inside and outside the greenhouse and energy consumption define objects and context. Sensors, actuators and processes and their relationship (context) are shown in Table 5. Context vector are (ID,time,date,GH1,relations,state) for each object/thing, where GH1 is the location ID of greenhouse.

All objects are interoperable using MQTT protocol. Publisher and subscriber communication model that implement this protocol allows interconnect all devices and things. Broker device are installed on fog node. Publishers and subscribers are implemented on different nodes.

### 4.3. Hardware Devices and Software Modules

Two edge nodes and one fog node are proposed to control climate and irrigation processes. Objects (things) and processes are deployed on all nodes. Irrigation and climate control are installed in edge nodes, AI services are implemented in fog node. Process control architecture is used in the first node type (edge) and data-centred architecture is used in the second node type (fog) and in the cloud services implemented. In edge node the flow of data comes from a set of variables (things and internal variables) which control the processes execution. Agronomist and expert users designs basic control algorithms. After learning and training process, these algorithms will be adjusted and modified following the results of the expert system (machine learning). The aim is to optimize resources (water, energy) without losing productivity. Two main control processes are executed in two edge nodes and one machine learning process is implemented in a fog node. Table 5 shows these processes and their relationship. Algorithms are implemented in Python and developed using open source criteria. Minimal hardware embedded devices requirements are shown in Table 6.

### 4.4. Installing and Testing

In facilities already automated, edge nodes are interleaved between the installed controllers and actual sensors and actuators. Some new sensors are installed to complete services designed (energy meter). In current facilities edge nodes are deployed and allows:

- Work in the same way as before (initial learning stage, analysis and model selection).
- Change to a new control using new expert and automatic rules using AI processes (supervised stage and training)
- Test and reconfigure expert rules (testing and maintenance)

**Figure 8:**
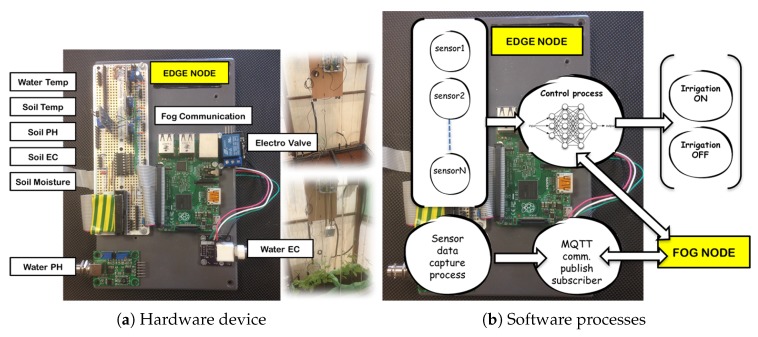
Hardware and software deployed on experimental greenhouse edge node.

Figure 8 shows an edge node to irrigation control and how is deployed in the experimental greenhouse built in this work, without previous installation. Agronomists and farmers preferences are those that drive the design: interfaces, maintain and optimize control processes. In irrigation process a time schedule with the selected flow rate is programmed by the user according to the period of crop growth. In learning stage, edge node captures the data and sends it to the fog node. Fog node process diary water uses, ambient and soil conditions, type of crop and its growth. Using these data crop type is classified. Crop production results are added as data to analyse it together with the stored ones. Production, water consumption, crop growing stage, time, date, soil parameters, current weather, forecast weather and ambient green house conditions are captured as inputs to machine learning platform.

In future crop productions the irrigation schedule can be automated, first with human supervision and then automated. Biophysical variables (plant, soil, canal flow, and weather conditions) that are measured during the growing seasons are used as inputs to build the models. Information about crop phenology (growth stages), soil moisture, and weather variables will be compiled. The analysis of irrigation decisions is important because this can help in the estimation of short-term irrigation demands. If the automated process decisions are known, It can help canal operators to better manage water deliveries and avoid unexpected delays and operational conditions that increase canal losses. Information about these demands can also be helpful for the evaluation of expected future agricultural supplies. It can never be possible to know the exact reasons why a farmer decided to irrigate, all farmers are different and prefer their own decision processes. Data analysis with farmer in in its own installation infer automated farmer actions. This data is used to build the models and these learnt frameworks will be used to predict irrigation decisions. The specific objectives planned with farmer are:

1. Identify the main variables contributing to an irrigation behaviour by training the models with relevant data
2. Group the irrigation decisions into distinct classes
3. Identify the decisions taken
4. Detect the patterns in farmer decisions
5. Infer future irrigation decisions using the information and modelling tools. Design decision tree algorithms to reduce water consumption

**Figure 9:**
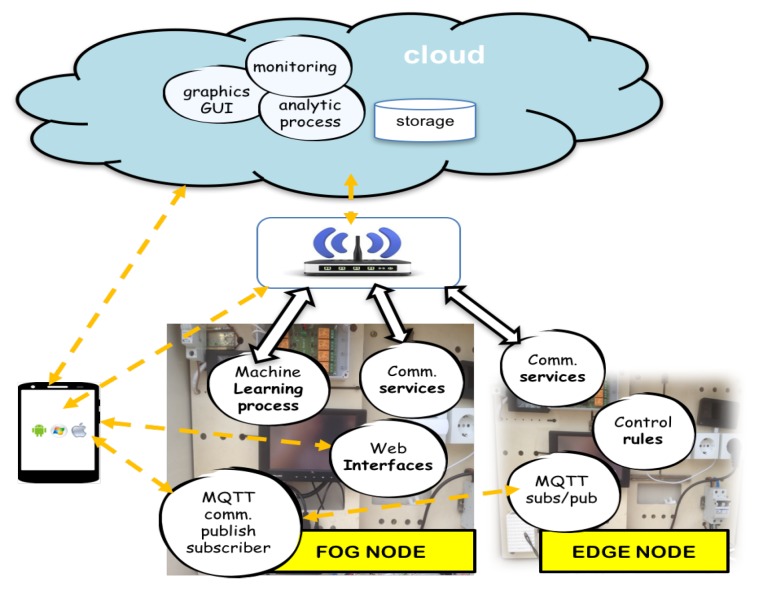
Communication and processes on edge and fog nodes.

Fog Computing node is shown in Figure 9. This paradigm extends the Cloud Computing to the edge of the network, thus enabling a new breed of applications and services. Defining characteristics of the Fog are: (a) Low latency and location awareness; (b) Wide-spread geographical distribution; (c) Mobility; (d) Very large number of nodes; (e) Predominant role of wireless access; (f) Strong presence of streaming and real time applications; (g) Heterogeneity [44].

**Figure 10:**
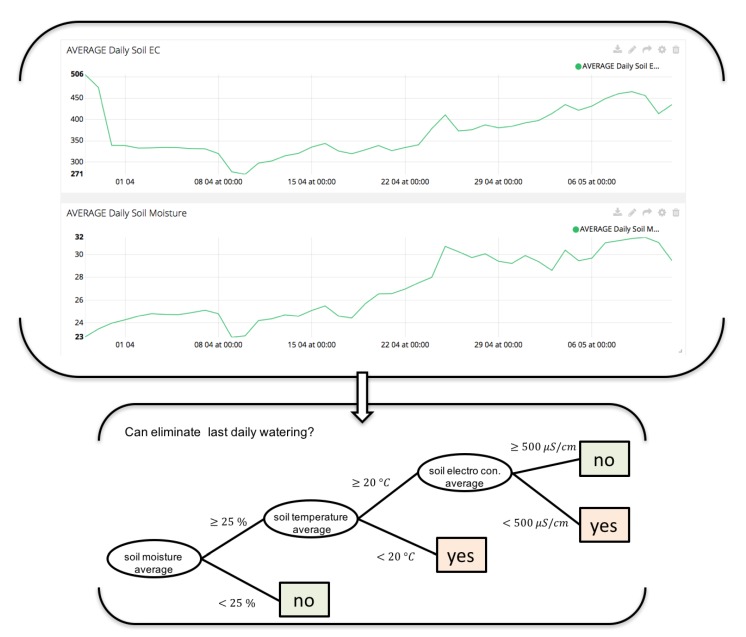
Decision Tree developed on irrigation control designed by agronomist and integrated on edge node. This decision tree aims to optimize water consumption. This new rule is designed by farmer observing the evolution of the data during the plant growth.

**Figure 11:**
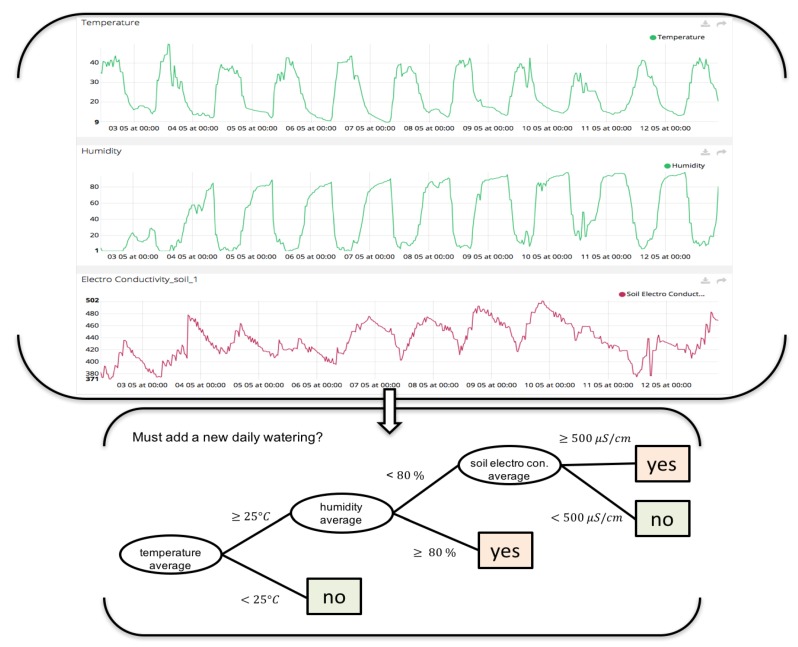
Decision Tree on irrigation control designed by agronomist and integrated on edge node. This decision tree aims to optimize plant growth control. This new rule is designed by farmer observing the evolution of the data during the plant growth.

In this paper, fog node is used to carry out the machine learning processes, storage data and communicate with cloud services (monitoring interfaces). Figure 10 and Figure 11 and shows different variables (soil moisture, soil temperature and conductivity means) that farmer decide to use in irrigation control to design new rules to optimize production. Before, control irrigation was controlled only by a time schedule. Now, it takes into account data sensors to decide if watering and growth control can be optimized with decision trees.

Cloud services designed are monitoring data accessed through a Human Machine Interface (HMI). IoT platforms push data from any Internet-Enabled Device and prompt them to quickly get started. Similar platforms with similar services show the state of commercial IoT technology: Azure [45], Ubidots [46], Thingspeak [47], are some examples of companies that provide IoT services. These platforms are built with similar architectures and provide, usually, the same resources: Application Programming Interface (API) communication between clients and IoT server.

**Figure 12:**
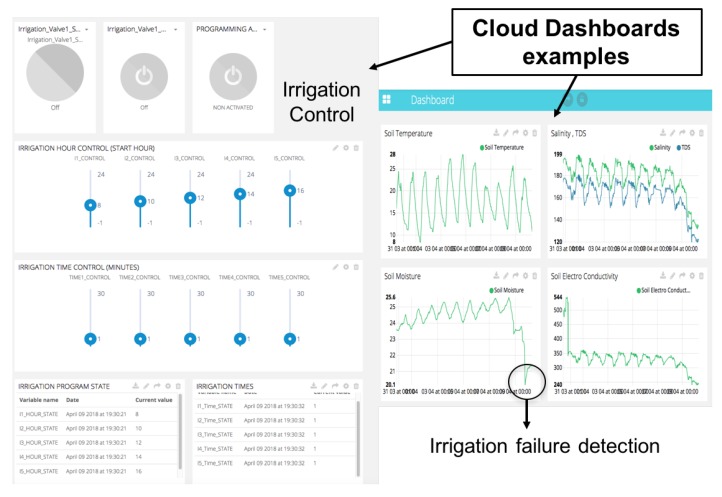
Dashboards designed for irrigation programming and monitoring.

All these platforms provide dashboard designs to monitor data using HMI formats pre-built. Using API services, processes in fog node are implemented to send new data to each dashboard. The API Documentation specifies the structure of the data that is exchanged between your devices and the Ubidots and Mobile-Alerts Cloud, along with code examples and libraries to speed up the project. Figure 12 shows a dashboard (interface HMI) designed by users on Ubidots cloud platform. The system can use standard protocols in the different layers and platforms that implement these protocols.

## 5. Conclusions and Future Work

In this work the state-of-the-art of PA and IoT technologies in agricultural scenarios, have been analysed. PA presents difficulties to be implemented by farmers. These include cultural perception, lack of local technical expertise, infrastructure constraints, knowledge and technical gaps and high start-up costs. Farmers must be involved in the design and integration of these technologies in their facilities. To carry out this solution there must be methods to facilitate such integration. This work proposes a new method to integrate the farmer in the development of new solutions using low cost sensing technologies and innovative communication paradigms. An architecture based on two new levels of communication and processing nodes (edge and fog nodes) form the technological core of the proposed method. Each level performs a set of interconnected functionalities. The proposed infrastructure can be installed either in already automated installations or in the design of new facilities. In the already automated installations, the method introduces new possibilities for the development of intelligent and interconnected control. An experimental work has been carried out in a greenhouse. In this work, communication nodes have been installed and a new service based on a decision tree paradigm has been designed by expert user. The facilities that use the proposed model make the climate control and irrigation subsystems interoperable and allow the farmer to design new integrated control rules. The new distributed communication model allows the farmer to analyse changes and improvements. This experimental work initiates a new methodology of work for the farmer who can use these new technologies more easily. Future control rules and services using a machine learning platform and AI paradigms will allow to optimize and improve the results.

## Figures and Tables

**Table 1:** IoT architecture: Layers.

Layers Proposed	Characteristics
3	Perception, Network, Application [7,8]
4	Things, Edge, Network, Application [9]
5	Business, Application, Service, Object abstration, Objects [10]

**Table 2:** IoT protocols.

Layer	Protocols
Session/Application	MQTT, CoAP, AMQT, HTTP, SOAP, ...
Network	6LowPAN, RPL, CORPL, IPSec, TCP/UDP, DTLS
Perception/Things	WiFi, Bluetooth Low Energy, Z-Wave, ZigBee, LoraWan, IEEE 802.15.4, LTE, ...

**Table 3:** Things context designed in smart control processes.

Object/Thing	Context
*Sensor${}_{i}$*	(ID,time,date,location,relations,state)
*Actuator${}_{i}$*	(ID,time,date,location,relations,state)
*Variable${}_{i}$*	(ID,time,date,location,relations,state)
*Process${}_{i}$*	(ID,time,date,location,relations,state)
*Controller${}_{i}$*	(ID,time,date,location,relations,state)

**Table 4:** Growth crop process on experimental station. Tomato plant growth stages.

lIrrigation actual (Total liters) = $5\;L/m^{2}$ (initial phase)		Average Temperature = $20{\;}^{\circ }C$
Energy used = 60 $\mathrm{Wh}/m^{2}/\mathrm{day}$		Average water PH = 6.5
Solar irradiance = $4\;\mathrm{kWh}/m^{2}/\mathrm{day}$ (NASA HOMER web)		Average water EC = 2850μS/cm
**Example of new service designed by user: Decision Tree to reduce water consumption**		
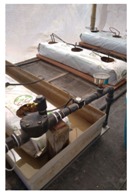	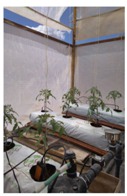	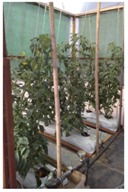
**Solutions**		**Technological results**
Develop a reference model based on distributed IoT paradigms New PA processes automated Graphics Interfaces use simple and universal access Tools, facilities and resources adapted for agronomist		GUI interfaces used on Internet New ways of data access and Low-cost deployment Users design DECISION TREE to optimize water consumption

**Table 5:** Things context identified in experimental greenhouse.

Object/Thing	Context	Type	Relation
*Soil${}_{temp}$*	$\left(ID_{1},time,date,GH1,{\;}^{\circ }C\right)$	input	$ID_{16},ID_{17}$
*Soil${}_{moisture}$*	$\left(ID_{2},time,date,GH1,\;\%\right)$	input	$ID_{16},ID_{17}$
*Soil${}_{PH}$*	$(ID_{3},time,date,GH1,\;$int)	input	$ID_{16}$
*Soil${}_{EC}$*	$\left(ID_{4},time,date,GH1,\;\mu S/{\mathrm{cm}}^{2}\right)$	input	$ID_{16}$
*Water${}_{temp}$*	$\left(ID_{5},time,date,GH1,{\;}^{\circ }C\right)$	input	$ID_{16}$
*Water${}_{PH}$*	$(ID_{6},time,date,GH1,\;$int)	input	$ID_{16}$
*Water${}_{EC}$*	$\left(ID_{7},time,date,GH1,\;\mu S/{\mathrm{cm}}^{2}\right)$	input	$ID_{16}$
*Inside${}_{temp}$*	$\left(ID_{8},time,date,GH1,{\;}^{\circ }C\right)$	input	$ID_{17}$
*Inside${}_{hum}$*	$\left(ID_{9},time,date,GH1,\;\%\right)$	input	$ID_{17}$
*Inside${}_{lum}$*	$(ID_{10},time,date,GH1,\;$lux)	input	$ID_{17}$
*Outside${}_{temp}$*	$\left(ID_{11},time,date,GH1,{\;}^{\circ }C\right)$	input	$ID_{17}$
*Outside${}_{hum}$*	$\left(ID_{12},time,date,GH1,\;\%\right)$	input	$ID_{17}$
*Outside${}_{wind}$*	$\left(ID_{12},time,date,GH1,\;m/s\right)$	input	$ID_{17}$
*Evalve${}_{water1}$*	$(ID_{13},time,date,GH1,\;$ON-OFF)	output	$ID_{16},ID_{17}$
*Evalve${}_{water2}$*	$(ID_{14},time,date,GH1,\;$ON-OFF)	output	$ID_{16},ID_{17}$
*Epump${}_{water}$*	$(ID_{15},time,date,GH1,\;$ON-OFF)	output	$ID_{16},ID_{17}$
*P_Irrigation${}_{1}$*	$\left(ID_{16},time,date,edge_{node1},\left[Ev_{1},Ev_{2},Ev_{3}\right]\right)$	process	$ID_{17}$
*P_Air_cond${}_{1}$*	$\left(ID_{17},time,date,edge_{node2},\left[M_{1},M_{2}\right]\right)$	process	$ID_{16}$
*Prediction${}_{weather}$*	$\left(ID_{18},time,date,GH1,\left[Water,Energy\right]\right)$	input	$ID_{16},ID_{17},ID_{19}$
*Forecast${}_{weather}$*	$\left(ID_{19},time,date,GH1,\left[Tem,Hum,Wind\right]\right)$	input	$ID_{16},ID_{17},ID_{18}$
*Energy${}_{meter}$*	$\left(ID_{20},time,date,GH1,\left[power_{measured},power_{predicted}\right]\right)$	input	$ID_{16},ID_{17}$
*HMI${}_{web}$*	$\left(ID_{21},time,date,webserver,\left[I/O\right]\right)$	interface	$\left[ID_{1},\ldots ,ID_{20}]\right)$
*P_Machine_Learning*	$\left(ID_{22},time,date,fog\_node,\left[management,supervision\right]\right)$	process	$\left[ID_{1},\ldots ,ID_{20}]\right)$
*Water${}_{flow}$*	$\left(ID_{4},time,date,GH1,\;m^{3}/h\right)$	input	$ID_{16}$

**Table 6:** Minimal hardware requirements of edge and fog nodes.

Requirement	Minimal Resources	Node Type
*Processor*	multi_core≥1000MHz	fog
*Processor*	core≥700MHz	edge
*Video*	GPU≥400MHz	edge
*Storage*	permanent≥1GB	fog
*Storage*	permanent≥600MB	edge
*Communication${}_{ports}$*	USB2.0,ethernet,WIFI	edge and fog
*Communication${}_{protocols}$*	serial,tcp/ip	edge and fog
*GPIO${}_{ports}$*	portI/O	edge
*Operating System*	Linux,Windows,OSx,Android,others	edge and fog
*Programming*	C,C++,Python	edge
*Programming*	C,C++,Python,web	fog

## Abbreviations

The following abbreviations are used in this manuscript:

AI	Artificial Intelligence
API	Application Programming Interface
HMI	Human Machine Interface
IAB	Internet Architecture Board
IETF	Internet Engineering Task Force
IoT	Internet of Things
ISO	International Organization for Standardization
ISOC	Internet Society
MQTT	Message Queuing Telemetry Transport
PA	Precision Agriculture
QoS	Quality of Service
RFC	Request for Comments
UCD	User Centred Design
UX	User Experience
